# Assessing vocal fold movement in healthy young children and infants using laryngeal ultrasound by speech and language therapists: a feasibility study

**DOI:** 10.3389/fneur.2026.1719167

**Published:** 2026-09-21

**Authors:** Joan K.-Y. Ma, Mari Viviers, Louise Edwards

**Affiliations:** 1 Clinical Audiology, Speech and Language Research Centre, Queen Margaret University, Edinburgh, United Kingdom; 2 Centre for Paediatrics and Child Health, Imperial College London, London, United Kingdom; 3 Department of Children’s Therapies, Imperial College Healthcare NHS Trust, London, United Kingdom; 4 School of Nursing and Midwifery, University of Lancashire, Preston, United Kingdom

**Keywords:** laryngeal ultrasound, paediatrics, speech and language therapy, vocal fold, vocal fold motion impairment

## Abstract

**Background:**

Vocal fold motion impairment (VFMI) is a common laryngeal disorder in infants and young children and may impact respiration, phonation and swallowing. Flexible nasendoscopy (FNE) is the current gold standard for assessment; however, its invasive nature may limit its use for routine screening. Recent studies reported good sensitivity and specificity of laryngeal ultrasound (LUS), a minimally invasive alternative compared to FNE. However, its application by Speech and Language Therapists (SLTs) in the paediatric population remains underexplored.

**Objectives/hypothesis:**

This study aimed to evaluate the feasibility of a novel SLT-led LUS protocol for infants and young children and to examine the reliability of defined laryngeal measurements.

**Methods:**

In this mixed-methods feasibility study, eleven participants (nine children aged 3- and 4-years-old and two 2-month-old infants) underwent an SLT-led LUS protocol incorporating quiet breathing and functional tasks. Completion and visualisation rates were analysed as an indicator of feasibility. Laryngeal landmarks were identified and laryngeal measurements were quantified in selected static images by two raters. Inter- and intra-rater reliability were evaluated using intraclass correlation coefficients (ICC). Inductive thematic analysis of the LUS operator’s field notes was used to identify experiences of conducting the protocol and areas for future refinement.

**Results:**

The protocol demonstrated high feasibility, with good completion and visualisation rates. Differences were noted across tasks, with a higher visualisation rate in quiet breathing and swallowing than in phonation. Inductive thematic analysis of the LUS operator’s experience highlighted areas for future refinement of the protocol. Quantitative analysis showed excellent reliability between raters across laryngeal measures, with moderate to excellent confidence intervals.

**Conclusion:**

This feasibility study provides initial evidence for an SLT-led LUS protocol to assess paediatric laryngeal function. LUS may enhance clinical pathways by enabling non-invasive screening and monitoring, while also serving as a research tool for phonation and swallowing. Further studies with larger and more diverse samples, including those with VFMI, are needed to establish clinical utility and generalisability.

## Introduction

Vocal fold motion impairment (VFMI) is a reduction or complete absence of movement of one or both vocal folds. It presents in the paediatric population due to reasons ranging from congenital anomalies, acquired laryngeal anomalies, birth trauma, neurological disorders, injury to the recurrent laryngeal nerve because of cardiac or aortic surgery, cervical and thoracic surgery, trauma and prolonged intubation. VFMI is one of the most prevalent laryngeal disorders in infants and young children (1, 2). The normal functioning of the vocal folds supports the regulation of airflow for respiration and phonation, as well as protecting against penetration and aspiration during swallowing. VFMI may impact swallowing, disrupt voice production (an altered or absent voice), lead to stridor and a weak cough. Changes in voice quality could negatively impact social communication and interaction for young children, affecting their quality of life (3). Speech and Language Therapists (SLTs) are uniquely placed to identify and support the communicative and psychosocial consequences of VFMI, in addition to the voice, swallowing and airway concerns that prompt referral in the first place. Flexible nasendoscopy (FNE) is currently considered the gold standard for assessing vocal fold movement and upper airway issues (4). However, in infants and young children, FNE can be difficult to carry out due to challenges in participation and the ability to manage this invasive procedure at a young age (4). Due to the risk of trauma and distress of FNE, sedation might be required in some circumstances (4, 5). In practice, this may mean the assessment is instead carried out as a microlaryngobronchoscopy (MLB) under general anaesthesia. This limits functional evaluation of vocal fold movement, specifically during phonation that is otherwise observed during an awake state. It also carries the additional risks associated with anaesthesia. The invasive nature and the associated challenges of FNE in paediatric populations limit its use for routine screening in high-risk groups, such as infants post-cardiovascular surgery, potentially delaying diagnosis and negatively affecting outcomes (2). In contrast, laryngeal ultrasound (LUS) is a less invasive, real-time imaging technique that has shown promising diagnostic accuracy in the paediatric population, with several studies reporting sensitivity and specificity between 78.5 and 100% in comparison with FNE (4, 6–10). These findings support the potential role of LUS as an adjunct tool in the assessment and management of VFMI.

In LUS, a standard B-mode ultrasound is generally reported as being used. Most studies also noted the use of a linear probe for laryngeal ultrasound [e.g., Sanchez-Jacob et al. (2), Zhang et al. (10), Alexander et al. (11), and Paprocki et al. (12)], and some included detailed information on the size of the probe, with a preference for a smaller footprint, such as the hockey stick transducer, in the paediatric population [e.g., Sanchez-Jacob et al. (2) and Lee et al. (7)]. Factors such as the size of the area of interest and the depth of the structure influenced the choice of the ultrasound probe. Given the relatively small size of the larynx and the anterior location of the larynx in the neck region, a high-frequency linear ultrasound was usually selected to maximise image resolution (13). A linear ultrasound probe emits ultrasound waves parallelly and therefore does not distort the resolution of the image in the near-field or far-field. In the application of LUS in the paediatric population, the ultrasound probe is placed transversely on the neck, generally at around the level of the acoustic window between the cricoid and thyroid cartilage, or through the non-calcified thyroid cartilage (1, 2). The laryngeal ultrasound image (Figure 1) is marked anteriorly by the thyroid cartilage, which is hypoechoic and can be easily recognised by its crescent shape. The arytenoid cartilages are also hypoechoic, marking the posterior boundary of the vocal folds. The anterior commissure is located directly posterior to the mid-point of the thyroid cartilage, with a hyperechoic appearance. Given that the false vocal folds cover the fatty tissue in the paraglottic space, they give off a more hyperechoic appearance than the true vocal folds, which cover the thin hypoechoic vocalis muscles. The visualisation of laryngeal structures in the paediatric population is relatively straightforward compared with adults, as infants and young children are not affected by the calcification of the cartilages seen in the ageing population, or the acute angle of the thyroid cartilage, particularly in the post-pubescent male population (14, 15). Hu et al. (13) conducted a large-scale study on true and false vocal folds visualisation rate using laryngeal ultrasound. They reported a 100% success rate in the visualisation of the laryngeal structures in participants under 18 years of age, regardless of gender. However, they noted a significant decrease in the visualisation rates of the vocal folds was observed in males (75 and 78.3% for true and false vocal folds for males versus 100% for both for females), with a gradual decrease along with the increase in age. The visualisation rates of males over 60 years of age were reported to be 38.1% for both true and false vocal folds.

**Figure 1:**
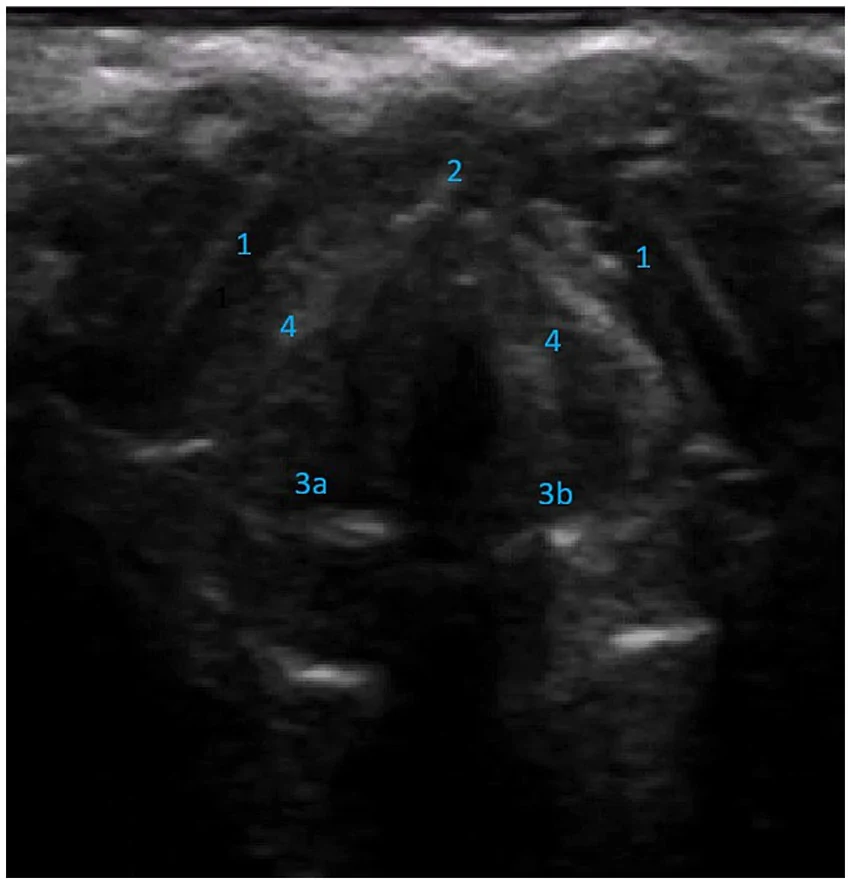
Laryngeal ultrasound image showing (**1**) thyroid cartilage, (**2**) anterior commissure, (**3**) right (**a**) and left (**b**) arytenoid cartilages and (**4**) false vocal folds.

The diagnosis of VFMI typically falls within the remit of Otolaryngologists, with Speech and Language Therapists (SLTs) routinely involved in managing the functional impact of VFMI in relation to swallowing and voice production. In the United Kingdom, SLTs do not routinely have access to instrumental assessment that enables direct visualisation of the vocal fold movement during routine clinical assessment. Instead, a referral to Otolaryngologists is made when a diagnosis of VFMI or changes in vocal functions are suspected by an SLT [RCSLT, (16); Smith and Sauder (17)]. This referral process could result in a delay in obtaining useful clinical information that could help inform SLT management of the infants’ and young children’s swallowing and voice needs. Therefore, the availability of LUS to SLT to screen and monitor changes in vocal fold function may help inform therapy approaches or prompt timely referrals to Otolaryngology colleagues. In recent years, there has been growing interest in the use of ultrasound by SLTs as a point-of-care tool in the assessment of swallowing and airway function (18, 19). While LUS protocol for vocal fold visualisation has been reported in the literature [e.g., Paprocki et al. (12) and Izadi et al. (20)], recent work has proposed SLT-specific protocols that extend beyond the assessment of vocal fold symmetry in quiet breathing to include functional tasks, such as sustained phonation (21, 22). Slinger et al. (22) published a LUS protocol specific to adult populations with inducible laryngeal obstruction, which included tasks such as the Valsalva manoeuvre and deep breathing. This highlighted the value of a clinical group-specific LUS protocol. Given the anatomical and behavioural differences in infants and young children, there is a need to develop and evaluate an SLT-led LUS protocol tailored to paediatric populations.

In comparing the diagnostic accuracy of LUS to FNE, most previous studies have focused on qualitative assessment, using dynamic LUS to obtain an overall impression of the symmetry of vocal fold movement by comparing the left and right sides (2, 4, 23), and commentary on the mobility of the vocal folds as being fully, partially or lacking any mobility (4). A few studies have attempted to quantify vocal fold movement using angle-based measurements (4, 12, 23, 24). Left and right vocal fold-arytenoid angle (VF-AA), together with the anterior glottic angle (AGA), were the most commonly reported LUS measurements. Ongkasuwan et al. (4) reported the VF-AA measurement to determine the predictive value of LUS compared with FNE in the identification of asymmetry or VFMI. In addition, they reported that a VF-AA value at maximum adduction that is less than or equal to 120 degrees is likely to be associated with asymmetry (sensitivity = 0.91; specificity = 0.68) (4). Paprocki et al. (12) provided an additional definition of the measurement points, describing the glottic angle as the angle formed by lines drawn from the posterior point of the anterior commissure to the medial margin of the arytenoids. The clarity of the definition of landmarks could be crucial to the reliability of measurements across assessors. Other reported measurements include the interarytenoid angle in maximum abduction (4), but the rationale for inclusion was not clearly defined. Instead of the arytenoid cartilage, Gao et al. (23) used the vocal folds as the key landmark for LUS measurement. They measured the angle between the medial margin of the vocal folds and the mid-sagittal axis of the larynx. In addition, they included distance measures of the laryngeal structures in their study, such as the distance of the vocal process to the midline and the distance from the anterior commissure to the arytenoid cartilages. In evaluating the differences between the qualitative and quantitative measurements, Sanchez-Jacob et al. (2) noted that in clinical practice of medicine, qualitative assessment of vocal fold symmetry using dynamic LUS is more practical and reliable than measuring VF-AA. It was reported that medical doctors do not have the time or the inclination to perform the VF-AA measurements (10). Instead, LUS are often interpreted qualitatively, similar to the assessment process of FNE (25). However, from the perspective of SLTs, quantifying VF movement to identify and monitor changes during the course of intervention and tracking variation in movement over time could be crucial.

### Aim

This feasibility study aims to lay the groundwork for the clinical implementation of an SLT-specific LUS protocol for assessing laryngeal function in infants and young children. Specifically, an SLT-led protocol tailored to infants and young children, which includes assessment of vocal fold movement in functional activities such as phonation and swallowing, would be evaluated. In addition, this study explored the reliability of a defined method of quantifying the laryngeal measurements during these activities by SLTs.

## Methods

### Study design

This mixed-methods feasibility study evaluated an SLT-led laryngeal ultrasound protocol for infants and young children. Quantitative measures were used to assess protocol completion, image visualisation and measurement reliability, while qualitative analysis of operator field notes explored experiences of conducting the protocol and identified areas for refinement.

### Participants

Eleven participants were recruited for this study, including nine 3- and 4-year-old children (four males and five females) and two 2-month-old infants (both female). Participants were recruited through university moderator messages, leaflets in the local community/supermarket bulletin boards, social media and the personal contact of the researchers. All participants had no reported history of vocal fold or airway issues, no previous experience of vocal fold examination, and no history of SLT intervention.

### LUS protocol development

Our current protocol was developed by the authors, who were all SLTs. The current protocol was established after consulting an existing unpublished paediatric protocol used by Otolaryngology colleagues at Birmingham Children’s Hospital (shared via personal communication) and a second protocol targeting adults with inducible laryngeal obstruction (22). Based on the extensive clinical experience in the paediatric populations of the authors, a range of functional activities critical to the assessment and management planning of SLTs, such as phonation and swallowing, were included to reflect on the professional practice.

### Ultrasound equipment

A Telemed HL 9.0/40/128z 40 mm linear ultrasound probe connected to the Echo B series ultrasound system running the standard B-mode ultrasound was used for data collection. The ultrasound probe has a frequency range between 5 and 10 MHz, a field of view (FOV) of 39 mm and 128 channels. The Echo B system was connected to a PC running Windows 11. The Telemed standard software, Echo Wave II, was used to capture the ultrasound recording in the session.

### Procedure

Data collection was carried out in the Ultrasound Recording Laboratory at Queen Margaret University. All ultrasound data recordings were conducted by the third author (LE), who has over 25 years of experience as a paediatric SLT. While ultrasound assessment is not part of standard SLT practice, all researchers had ultrasound training through attending head and neck ultrasound training courses, informal training, supervision, such as learning from Otolaryngologists and a specialist SLT with extensive vocal fold knowledge and ultrasound experience, hands-on practice with both school-aged children and adults, and through participation in the International SLT Ultrasound working group.

To facilitate engagement and understanding among the young participants (specifically the 3- and 4-year-olds), a stop-motion animation was created outlining the data collection procedure.^1^ All participants had access to the link prior to the data collection and were shown the video on the day to prepare them for the procedure and offer a chance to ask any questions. In addition, a toy ultrasound probe, a baby doll, a PlayMobil™ ultrasound set, and other toys and rewards commonly used in paediatric speech and language therapy sessions were available in the recording room to support and encourage participation during the session.

Data collection for the 3- and 4-year-olds was conducted in an upright position, with the child either sitting independently or on the lap of their parent. For the infants, the procedure was carried out in the supine position, except for an incidence of recording during breastfeeding, which was recorded with the infant in the arms of the mother in a cradle hold position. The SLT researcher began by placing the ultrasound probe anteriorly at the level of the thyroid cartilage. The data collection session commenced once a clear vocal fold image was established. For the children, the following activities were observed on ultrasound, beginning with quiet breathing, followed by either a prompted dry swallow or cup drinking. For the speech tasks, the children were asked to imitate a range of speech sounds together with visual prompts, including three vowels (/u/, /i/ & /a/) and three consonants (/z/, /s/ & /m/). Counting was also attempted if it appeared appropriate. A cineloop was recorded for quiet breathing and for swallowing individually. For the speech tasks, one cineloop was recorded for each successful phonation of the target sounds. For the infants, the movement of the vocal folds was recorded during quiet breathing, vocalisation (including crying) and swallowing (e.g., spontaneous swallow and swallow during drinking). Given the young age of the infants, these recordings were opportunistic rather than in a planned sequence. An audio recording of the entire session was also carried out to support data analysis.

Field notes were also documented at the end of each session to record observations, experiences and insights of the third author (LE) in conducting the data collection. These reflexive accounts support trustworthiness and clarity of the qualitative data reported in the study.

### Data analysis

The feasibility of the LUS protocol was evaluated by the completion rate per protocol component and the visualisation rate. The completion rate referred to the percentage of participants who successfully performed each task (quiet breathing, swallowing and phonation). The counting trials were discounted from the analysis because they were not consistently attempted by all participants. Each LUS recording was then qualitatively assessed to determine if the key anatomical landmarks (thyroid cartilage, anterior commissure and left and right arytenoids) could be simultaneously identified within a single frame. The visualisation rate was defined as the proportion of recordings in which all anatomical landmarks were identifiable together within the same frame. In addition, the percentages of successful observations of symmetrical movement in functional swallowing and phonation were also calculated based on dynamic vocal fold movement observed throughout the task, rather than a single static frame. The image quality was evaluated jointly by the first and second authors (JM & MV). As all the participants had no history of vocal fold pathology, symmetrical vocal fold movements were expected and confirmed by a clinical observation of the third author (LE) at the time of data collection.

An inductive thematic analysis of the field notes was conducted to critically evaluate the data collection protocol and identify areas for further refinement. The first author (JM) read the field notes and labelled observations into broad categories. Similar observations were grouped together, and recurring patterns were used to develop five themes inductively. The field notes were then revisited to refine the themes and identify relevant observations to support each theme (26).

Quantitative analysis was used to examine the reliability of SLTs in quantifying the laryngeal measurements. A set of ten static images was extracted and used as test stimuli for the two raters to calibrate their ratings against each other and with two expert raters (an Otolaryngologist and a specialist SLT). A separate set of fifty static images, including five from each child (9 children x 5 images each = 45 images) and an additional five images from the two infants, was extracted by the first author from the cineloops to represent a range of VF angles. Images were purposively selected to capture the clearest available frame representing full abduction, partial abduction and adduction for each participant. Of the 50 images, 14 represented full abduction (28%), 14 represented adduction (28%), and 22 represented partial abduction (44%). Ten images (20% of the static images) were repeated for intra-rater reliability measurement, resulting in a total of 60 images. All images were randomised and assigned a unique image number to ensure blinded measurement. Additionally, a four-week gap was included between the extraction and measurement to minimise exposure bias. The first and second authors each independently rated sixty images. The left and right VF-AA and the anterior glottic angle of each image were measured using the angle tool on ‘ImageJ’ (27). The measurement approach was adapted from those outlined by Ongkasuwan et al. (4) and further refined. To measure the VF-AA, two lines were drawn: the first extended from the most anterior point of the anterior commissure to the most medial point of the corresponding arytenoid; the second was aligned along the posterior edge of the arytenoid. The AGA was calculated between the two lines extending from the anterior commissure to the medial aspect of each arytenoid (See Figure 2). The inter- and intra-rater reliability was compared by the Intraclass correlation coefficient (ICC) using a random two-way absolute agreement model on IBM SPSS Statistics for Windows, version 23 (IBM Corp., Armonk, NY, USA). ICC values and the 95% confidence interval (CI) of the ICC estimates were interpreted following the criteria outlined by Koo and Li (28), with < 0.5 indicative of poor reliability; 0.50–0.75 moderate reliability; 0.75–0.90 good reliability; and > 0.90 representative of excellent reliability.

**Figure 2:**
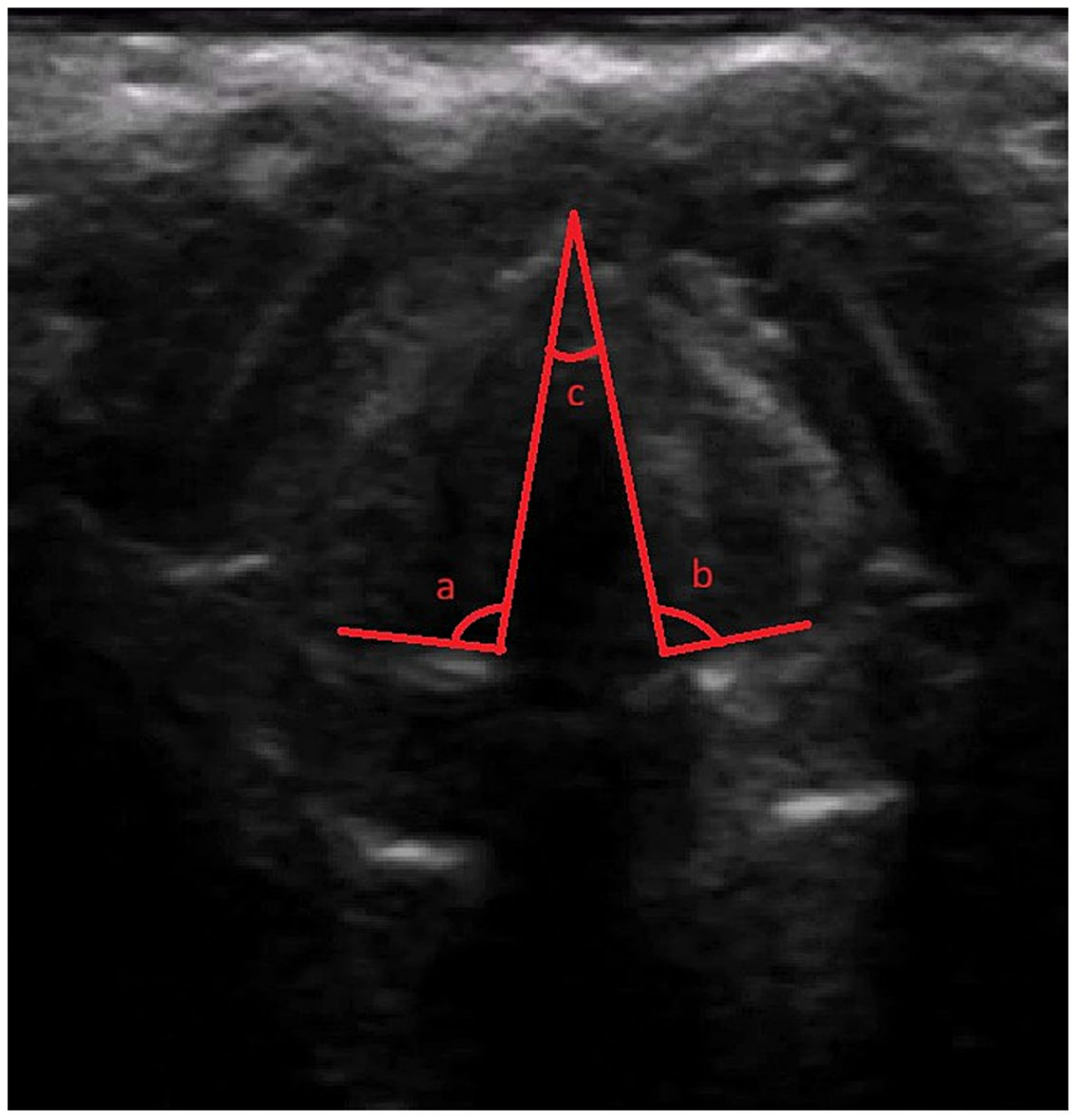
Laryngeal ultrasound measurement (**a**) right VF-AA, (**b**) left VF-AA and (**c**) AGA.

## Results

### Protocol evaluation

The protocol completion rate was calculated separately for young children and infants due to the differences in their ability to engage with the examiner. For the young children group (3- and 4-year-olds), LUS recordings were successfully obtained in eight out of nine (89%) and seven out of nine (78%) of the participants during quiet breathing and swallowing, respectively. LUS images were recorded for all participants in phonation tasks, with six out of nine participants (67%) completing the vocalisation of all six sounds (three vowels and three consonants), and the remaining three participants (33%) completing only three out of the six phonation tasks. For the two infants, LUS recordings during quiet breathing and phonation (vocalisation or crying) were successfully obtained for both participants, with swallowing images recorded for one of them. The protocol completion results are summarised in Table 1.

**Table 1:** Protocol task completion by participant.

Participant	Age	Quiet Breathing	Swallow	Phonation (sounds completed/total trials)
P1	4	✓	✓	6/6
P2	3	✗	✗	6/6
P3	3	✓	✓	3/6
P4	4	✓	✗	3/6
P5	3	✓	✓	6/6
P6	3	✓	✓	6/6
P7	3	✓	✓	3/6
P8	4	✓	✓	6/6
P9	8 weeks	✓	✗	Crying and vocalisation
P10	3	✓	✓	6/6
P11	9 weeks	✓	✓	Crying and vocalisation

The anatomical landmarks, including the thyroid cartilage, anterior commissure and arytenoid cartilages, and vocal fold movement were clearly identifiable in at least one LUS image for all the participants. For quiet breathing, the anatomical landmarks and symmetrical vocal fold abduction were observed in 8 out of 9 (89%) of the cineloops for the young children and 2 out of 2 (100%) of the cineloops for the infants. For swallowing, the anatomical landmarks and vocal folds approximation were observed in 100% of the cineloops for both the young children (7 out of 7) and the infants (2 out of 2). In the phonation tasks, 45 cineloops were recorded from the young children, and five from the infants. For the young children, anatomical landmarks and clear vocal fold movements were observed in 80% of the recordings (i.e., 36 out of 45 cineloops), with only the right side of the vocal folds being observable in 4% (i.e., 2 out of 45 cineloops). In the remaining 16% (i.e., 7 out of 45 cineloops), no clear vocal fold movements could be observed. For the infants, anatomical landmarks and clear vocal fold movements could be observed in 100% of the data (i.e., 5 out of 5).

#### Qualitative analysis

Inductive thematic analysis of the field notes identified five key themes relevant to conducting the LUS protocol: participant age and behaviour, task challenges, positioning, ultrasound equipment and technical considerations, and overall user experience. Illustrative excerpts from the field notes are included within each theme to support the interpretation of the findings.

##### Theme 1: Participant age and behaviour

All participants in our study generally tolerated the LUS procedure well, though individual differences in engagement were observed. The young children showed variable levels of participation, with some described as “incredibly shy but able to participate” or “really reluctant to participate”, while others engaged readily with encouragement or rewards such as stickers. The two infants in the current study both tolerated the procedure calmly, particularly when scanning occurred during or after crying. In one instance, the application of the ultrasound probe subjectively appeared to settle a crying infant.

##### Theme 2: Task challenges

Mixed responses were observed in the phonation tasks, with substantial encouragement required to elicit phonation from some children, with whispered responses or selective participation observed. In one session, the child “refused to do practice run-through of sounds initially and then when gave them a go, often whispered them.” However, some children engaged by contributing spontaneous responses and preferred words; one child “added some sounds/sentences he wanted to say” but declined counting. Practising the sounds before probe placement appeared to support better engagement in some children, with one child noted to have “benefited from practising the sounds beforehand.” Swallowing tasks could also be challenging to capture due to reluctance, with one session noting that “swallowing and single sounds” were missed “due to reluctance.”

##### Theme 3: Positioning

The positioning of participants had an observable impact on image capture. Many children were seated on their parents’ laps, and loss of probe contact was common due to slouching, chin tucking, or head turning towards parents or other external stimuli. For example, one field note described “turning in mum’s lap [leads to] loss of probe placement,” while another noted that the child was “tending to slump back on mum’s lap so contact with neck lost and impacting on images.” This was particularly evident in younger children who required more postural support to maintain optimal alignment. In infants, more flexible positioning (e.g., in arms, side-lying) was possible, though frequent head movement necessitated readjustment.

##### Theme 4: Ultrasound equipment and technical considerations

Adjusting frequency, depth, or gain was not possible while actively recording, and there were instances where saving and labelling recordings disrupted the scanning process, leading to loss of probe position. This was described in the field notes as “fiddly-ness of saving the recording and labelling the file whilst maintaining probe position.” Although a separate audio recording was made of the session, the audio recordings were not synchronised to the ultrasound recording. There were also cases where vocalisation was not successfully captured within the cineloop, despite being produced during scanning. Technical difficulties were also encountered with the recording system, which “crashed part way through” one session. These issues highlight the need for streamlined software control or staffing arrangements to support simultaneous scanning and data management.

##### Theme 5: Overall user experience

The ultrasound procedure was generally well-accepted by both children and their caregivers. Most children described the gel and scanning experience using positive or imaginative sensory language, such as “squishy”, “wet”, “like yoghurt”, or “like fish”. One child associated the gel with food when the term “jelly” was used, underscoring the importance of choosing developmentally appropriate and unambiguous language when describing the procedure. Parents generally reported that the procedure was non-distressing, including for children whose parents had noted sensory sensitivities. Many parents commented positively on the procedure as non-invasive and easy, with several expressing surprise at their child’s willingness to participate. Visual resources such as the stop-motion animation and illustrated leaflets were viewed as helpful in preparing families and supporting understanding of the procedure.

### Laryngeal measurement

Inter-rater reliability of the three laryngeal measures (left VF-AA, right VF-AA and the AGA) was compared independently between the two raters across the 50 static ultrasound images. Intra-rater reliability for the same measures was also calculated by repeating 20% of the analysis. The ICC showed excellent inter-rater reliability for all three laryngeal measures (ICC = 0.92–0.97, 95% CI 0.86–0.98; see Table 2). For the intra-rater reliability, good to excellent intra-rater reliability was observed for left and right VF-AA (95% CI 0.77–0.99), and moderate to excellent reliability for the AGA (95% CI 0.70–0.99; see Table 3).

**Table 2:** Inter-rater reliability analysis for the three laryngeal measures [left and right vocal fold-artenoid angle (VF-AA) and anterior gottic angle (AGA)] using intraclass correlation coefficient (ICC).

Laryngeal measurement	ICC	95% CI
Left VF-AA	0.95	0.92–0.97
Right VF-AA	0.92	0.86–0.95
AGA	0.97	0.95–0.98

**Table 3:** Intra-rater reliability analysis for the three laryngeal measures using ICC.

Laryngeal measurement	Rater 1		Rater 2	
	ICC	95% CI	ICC	95% CI
Left VF-AA	0.94	0.77–0.98	0.94	0.77–0.99
Right VF-AA	0.95	0.80–0.99	0.95	0.81–0.99
AGA	0.92	0.70–0.98	0.96	0.83–0.99

## Discussion

The first aim of this study was to evaluate the feasibility of an SLT-led LUS protocol for infants and young children, with a particular focus on the visualisation of vocal fold movement in functional activities. Overall, there was a high completion rate for the LUS protocol in both groups, with higher completion rates noted for quiet breathing and swallowing compared to phonation tasks. These differences could be related to several variables. First, the sequence of the task could have contributed to the differences, as the current protocol began with quiet breathing and swallowing, and progressed to phonation. Task effect has long been observed in cognitive studies in children (29), affecting the children’s performance on individual tasks. This could be related to the level of sustained attention development, which was found to be rapidly developing up until the age of ten (30). Second, task complexity could have impacted the level of engagement. Phonation tasks placed greater cognitive and behavioural demand on the children. Unlike breathing and swallowing, which are automatic and familiar activities, the phonation of specific sounds may appear novel and more challenging. For example, it was observed that some children preferred spontaneous utterances to the structured phonation task. Third, contextual factors, such as shyness or distraction during scanning, may have further influence on the completion of the phonation tasks.

A high visualisation rate was observed in the LUS recording, with successful visualisation of anatomical landmarks and vocal fold movement in at least one of the recordings for all participants. While the high visualisation rate was positive, especially considering the dynamic nature of the tasks included, it also highlights some of the inherent challenges in LUS in young children and infants. The lower visualisation rate of the phonation task could be linked to the number of trials, including three vowels, three consonants and a counting task in some cases. These required good attention and sustained engagement from the children. In addition, there is a larger range of laryngeal movement in phonation and swallowing and the challenges in maintaining probe stability during the tasks. For example, only one side of the vocal folds was visible on 4% of the data, suggesting issues in the alignment of the probe with relevant anatomic landmarks. Other operator-related variables, such as experience, are known to contribute to visualisation rate and reliability on different types of ultrasound (31, 32). This also reflected the challenges of having only a single operator in our current protocol, who was responsible for scanning, recording and also engaging the child.

In comparison to most previous protocols in which the children were scanned in supine position [e.g., Lee et al. (21), Ongkasuwan et al. (4), Wang et al. (24)], an upright position was adopted in this feasibility study to create a naturalistic and functional context and to reduce the medicalisation of the procedure. Although image acquisition and visualisation might be more challenging in an upright position, this choice aligns with the clinical rationale of the current protocol in SLT practice, particularly for functional tasks such as swallowing and phonation. Direct quantitative comparison with supine protocols was not possible, as completion and visualisation rates are not consistently reported in a comparable format across existing studies. However, the rationale for upright positioning in the current protocol was primarily driven by the aim of assessing vocal fold movement during natural, functional activity, particularly swallowing and phonation, rather than by an assumption that upright positioning offers superior image quality. Given that the current study achieved good overall visualisation rates in this position, we would argue that the functional benefits of upright positioning are a reasonable basis for its use, provided visualisation remains adequate. The comparative effect of positioning (upright, semi-supine, supine) on LUS image quality and functional task performance is an important question that warrants dedicated investigation, rather than as a secondary consideration within a broader feasibility study.

The high overall completion and visualisation rates indicated that the core elements of the current protocol are workable, but the differences in performance across tasks and reflection on experience suggest that fine-tuning might be required in optimising its clinical implementation. The phonation task presented the most significant difficulty in the current protocol. Future iterations could explore reordering the tasks and introducing phonation earlier, reducing the number of phonation trials by selecting the ones with the most contrastive VF movements (e.g., an /i/ vs. /a/ contrast, reflecting greater and lesser degrees of vocal fold adduction), or dividing them into shorter subtasks. Practising the sounds before beginning the recording might also be helpful in some cases. Clinically, the lower success rate for phonation suggests that a smaller number of carefully selected, contrastive tasks may be more practical and informative than a comprehensive set of trials in routine use. It is also worth noting that ongoing technical developments in ultrasound systems, including improved capability for synchronised audio-visual recording, are likely to further support the reliable capture and evaluation of phonation tasks in future applications of this protocol. Practical refinements concerning image capture are also needed. A two-person set-up, with one focusing on image acquisition and one engaging the child with the task, might help to improve image acquisition reliability. Lastly, exploration of different child-friendly seating arrangements, such as chairs with a high back or head support, might address the challenges of probe contact due to head movement during image acquisition.

The overall experience of LUS for both the children and their parents was positive. All children attempted at least part of the procedure, with the majority completing the full protocol. Parents expressed positive experiences and surprise at the level of child participation. LUS was reported as neither distressing nor invasive by the parents. While the inherent less invasive nature of LUS and the naturalistic task demands of the current protocol were likely to contribute to the positive reception, the acceptability of the LUS procedure was also shaped by the way it was delivered. The researcher conducting the data collection was an experienced paediatric SLT with expertise in engaging with children, and considerable preparation was included to optimise the child’s experience. Fear of the unknown generally contributes to the anxiety of medical procedures and negative experiences, and a clear agenda and expectation could support patients to feel more in control and reduce anxiety (33). Szeszak et al. (34) showed that providing an animation of an imaging procedure (i.e., MRI) in advance of the procedure helped reduce the anticipated anxiety and improve understanding of the expectation of the procedure. In line with this, a stop-motion animation was developed in our study, as an outcome of our engagement with school-aged children, to introduce the LUS procedure. It was shared with all the child participants and parents prior to the procedure. In addition, elements of pretend medical play were also used in preparing the children, including a toy ultrasound probe, a toy ultrasound machine and a doll. The availability of medical play can help to normalise the experience of the procedures and allow the children to express their fears and misconceptions (35), which could be addressed by the researcher to improve the engagement and experience. Additionally, positive reinforcement via traditional rewards, such as stickers and toys, were also used to maintain motivation and engagement throughout the session. The combined non-invasive nature of the LUS procedure and the use of child-centred engagement strategies contributed to the high level of acceptability observed in the current study, which are essential to be considered in the future implementation of LUS in clinical SLT practice.

The second aim of this study was to explore the reliability of a defined method of quantifying the laryngeal measurements during functional activities. The results of the current study demonstrated excellent inter- and intra-rater reliability, with confidence intervals ranging from moderate to excellent. This showed promising results of SLTs carrying out laryngeal measurements in LUS. While LUS measurements were generally described as easy to learn (11, 36), a few factors could have contributed to the high reliability in the current study. A recent study reported significant improvement in laryngeal measurements after training among SLTs (21). A stimulus–response-feedback-stimulus (SRFS) paradigm was used in the study by Lee et al. (21) to support the development of an internal standard of LUS image and differentiation of vocal pathologies. This included the use of ‘expert measurement’ as the gold standard in the design of feedback. Although the two raters in the current study did not undergo a formalised training programme, they gained experience in LUS image interpretation through a series of continuous professional training. Similarly, the raters had compared their own labelling of the training images to the expert raters (an Otolaryngologist and a specialist SLT), supporting the development of internal consistency in the labelling of laryngeal structures. Additionally, the refined definition of the laryngeal measurements in the current study also contributed to the good reliability. The two raters spent dedicated time calibrating the definition and measurement techniques with the test stimuli prior to the main study. One of the primary sources of differences was in the labelling of the arytenoid, which is traditionally described as triangular, and its anterior and posterior edges provide different potential landmarks. Alignment along these alternatives could produce variation in VF-AA measurement. To resolve these differences, the raters had undergone an iterative consensus-building process with a separate set of test LUS images, which led to a refined and shared operational definition of the measurement landmarks. For example, VF-AA is measured by the intersection between the arytenoid marker along the posterior edge of the arytenoid and a second line linking the most anterior point of the anterior commissure to the most medial point of the arytenoid. Therefore, clear operational definitions of LUS measurements, good quality continuous professional development and appropriate expert support will be crucial considerations in the future skill development for LUS image acquisition and measurement.

### Limitations and future directions

Several limitations to this study should be considered when interpreting the findings. First, the current study has a small sample size, with only nine young children and two infants, and no participants with VFMI were included. While this provided valuable preliminary data on the feasibility of the protocol, a larger sample size with children across different age groups, and the inclusion of children with and without VFMI would be required for a comprehensive evaluation of the protocol. This would help clarify whether protocol completion and visualisation rates are maintained, as children with prior medical experience could be more settled with handling, and others more wary. Second, the image acquisition was conducted by a single operator in this study. This setup ensured consistency across recordings, but the completion rate and visualisation rate might have been impacted by the demand of managing the technical aspects of the ultrasound system, audio recording and keeping the participant engaged. Future studies exploring the reproducibility of the protocol completion rate and the visualisation rate across multiple operators and different setups are required. Thirdly, the reliability analysis was conducted by two raters who worked closely together in refining the measurement definitions. While this collaborative process likely enhanced internal consistency, it may have also inadvertently reduced rater independence and introduced subtle consensus bias. A larger pool of raters working independently using the definition derived from this study could confirm the reproducibility of the laryngeal measurement reliability. Additionally, the application of machine learning to the tracking of arytenoids and the anterior commissure could offer an alternative to LUS quantification. A similar application has already been developed for the tracking of the hyoid movement on ultrasound for swallowing evaluation by SLTs (37). Fourthly, the current analysis was restricted to the laryngeal measurement of the static images. The variability in image selection, such as the frame representing maximum adduction, was not explored. The measurement of maximum adduction could be crucial for the clinical application of LUS for SLT to support the monitoring of changes in VF movement. Frame selection is generally done by visual scanning of the ultrasound recording to identify the desired parameters, such as maximum adduction, which is highly subjective. A future study to explore the reliability in frame selection is required to ensure the validity and reliability of LUS measurements. Finally, the lack of synchronised audio channels to the ultrasound made it challenging to link the VF movement to a specific functional phonation activity. While most ultrasound systems do not have a synchronised audio channel, such a system has been developed in the area of submental ultrasound in the study of speech sound production [e.g., Cleland et al. (38)]. Future technical development of an ultrasound system with the option of synchronised audio and ultrasound recording would enhance the application of LUS in speech and language therapy.

## Conclusion

This feasibility study demonstrated that an SLT-led LUS protocol tailored for infants and young children can be successfully implemented with high completion and visualisation rates. Through training and operationally defined measurements, a high level of reliability was achieved for the quantification of laryngeal measurements. These findings provide encouraging preliminary evidence for the clinical translation of LUS as a practical and informative tool for SLTs in the assessment of laryngeal function. Furthermore, there is potential for LUS to be used as a research tool in advancing the study of phonation and swallowing in the paediatric population. The minimally invasive nature of LUS and its ease of use through structured training make it a valuable addition to the multidisciplinary pathway for the evaluation of VFMI. Wider clinical adoption may improve access, enhance care experiences for children and their families, and facilitate more timely contributions from SLTs alongside the diagnostic process of otolaryngology pathways. Parallel to the investigation of the feasibility of the LUS protocol, studies exploring the perspectives of the parents and SLTs on the acceptability and perceived value of LUS were carried out. Taken together, this body of work contributes to the development of a novel clinical pathway for SLT-led LUS in infants and young children, supporting its future integration into both clinical practice and research.

## Data Availability

The raw data supporting the conclusions of this article will be made available by the authors, without undue reservation.

## Footnotes

1
https://tinyurl.com/Voicebox4Kids
